# Chemotherapeutics for *vivax* malaria

**DOI:** 10.1186/1475-2875-11-S1-O21

**Published:** 2012-10-15

**Authors:** J Kevin Baird

**Affiliations:** 1 Eijkman-Oxford Clinical Research Unit, Jakarta, Indonesia

## 

*Plasmodium vivax* imposes significant burdens of morbidity and mortality across the malaria endemic world. Treatment of this infection requires a blood schizontocide against the acute attack and hypnozoitocide against relapses. Chloroquine combined with primaquine has been the therapy of choice for radical cure of *vivax* malaria since the 1950s. Primaquine, however, was never optimized or adapted for use in endemic zones and its toxicity in prevalent G6PD-defkient patients (typically 5-20%) sharply limits its effectiveness. Resistance to chloroquine has emerged in Southeast Asia and now threatens the Indian sub-continent where most *P. vivax* occurs. The research community faces the steep challenge of developing new radical cure strategies. This presentation explores those challenges and the means to meet them, principally optimizing primaquine as a partner to new ACTs for maximum efficacy and more practical dosing and safety.

